# PReS-FINAL-2287: Electroconvulsive therapy in a patient with juvenile systemic lupus erythematous

**DOI:** 10.1186/1546-0096-11-S2-P277

**Published:** 2013-12-05

**Authors:** BE Bica, LMS Rivera, AN Nicol, GS Campos

**Affiliations:** 1 Rheumatology, Universidade Federal do Rio de Janeiro, Rio de Janeiro, Brazil

## Introduction

Patients with systemic lupus erythematous (SLE) often develop neuropsychiatric disease. Central nervous system (CNS) manifestations of SLE occur in about half of all patients, and psychiatric presentations are seen with a prevalence of 35% to 60% in this group. Electroconvulsive therapy (ECT) also known as electroshock therapy is a psychiatric treatment in which electricity/electric shock is used to induce convulsions. It is used to treat psychiatric diseases such as depression, schizophrenia, mania or catatonia that do not respond to conventional treatment.

## Objectives

To describe a patient with refractory neuropsychiatric lupus who responded to electroconvulsive therapy.

## Methods

Case Report: A 25-year-old woman with a 13-year history of SLE characterized by arthritis, thrombocytopenia, positive antinuclear antibody in a titer of 1:2,560 (speckled pattern), and the presence of antibodies to Sm and antiribosomal P protein autoantibodies was admitted to University Hospital with an organic psychosis. She was treated with corticosteroids, cyclophosphamide and azathioprine and went into remission. When she was 18 yo, she had a relapse (depression) and was treated with methyl prednisolone (MP) pulse therapy (PT) and cyclophosphamide. After 6 months, she had another episode of psychosis (echolalia, repetitive hands movements, extreme anxiety and suicide tendency) which did not respond to MP PT, Cyclophosphamide, Rituximab and Intravenous Immunoglobulin. Magnetic Resonance of the brain was normal, CSF results were inconclusive, antiphospholipid, anti dsDNA and anti Sm antibodies were all negative. She had various episodes of aggressiveness, agitation, insomnia, nightmares, hallucinations, difficulty in concentrating, repetitive movements of both upper and lower limbs, phases of catatonia and suicidal tendencies. Psychotropic agents, antidepressants and anxiolytics were used with little response clinically. After 18 months of immunosuppressive and anti-psychotics treatment without improvement, an ECT trial was begun. After 4 sessions of ECT, the patient had a remarkable improvement of her psychiatric symptoms, she was able to sleep and respond normally to her surroundings. She had a total of 10 sessions of ECT.

## Results

Psychosis is one of the severe neuropsychiatric manifestations of LES. There are few studies about the use of electroconvulsive therapy in the treatment of neuropsychiatric lupus. Mon, L'Ecuyer et al (2012) reported the use of ECT in the treatment of a child with catatonia and neuropsychiatric lupus with similar good results.

## Conclusion

The failure of antipsychotic and anticonvulsant medications, benzodiazepines, high-dose steroids, rituximab and immunoglobulin in this patient dictated the need for other treatment modalities. The decision to use ECT was based on the success of this treatment for psychiatric manifestations of systemic disorders. There are few reports about the experience of ECT in psychiatric manifestations of SLE.

## Disclosure of interest

None declared.

